# Phospholipase Cβ regulates negative associative memory through calcium dependent and independent mechanisms

**DOI:** 10.1016/j.jbc.2026.113308

**Published:** 2026-06-29

**Authors:** Pingjin Chu, Madison Rennie, Suzanne Scarlata

**Affiliations:** Department of Chemistry and Biochemistry, Worcester Polytechnic Institute, Worcester, Massachusetts, USA

**Keywords:** C. elegans, calcium signaling, CREB, Early growth response protein-1 (EGR-1)learning, memory, Phospholipase Cβ

## Abstract

The Gαq/PLCβ signaling plays a key role in learning and memory. In mice, the loss of PLCβ1 results in inappropriate responses to adverse events that is directly tied to the ability of PLCβ to mediate calcium signals. Besides this calcium-mobilizing function, PLCβ1 has been shown to have a cytosolic, activity-independent function that keeps stress granules disassembled until stimulation and mediates the nuclear localization of the transcription factor Early Growth Response −1 (EGR-1). Here, we assessed the impact of PLCβ on memory formation in the nematode, *Caenorhabditis elegans*, whose Gαq/PLCβ signaling system is analogous to mammalian systems. Wild-type N2 worms and *egl-8^−/−^*mutant worms that lack PLCβ were studied using a negative associative memory paradigm involving subjecting them to an attractive odor while simultaneously starving them. We find that wild-type worms have a significantly lower attraction to the odor than controls, while *egl-8^−/−^*worms showed a more pronounced response, supporting a role of PLCβ in long term adverse reaction behavior. Using immunostaining, we find that the *egl-8^−/−^*mutants displaying strong memory formation had higher CREB levels and lower nuclear EGR-1. To better delineate the roles of PLCβ1 on memory, we overexpressed active and inactive PLCβ1 in PC12 cells. Increasing cellular PLCβ1 reduces CREB levels in accord with the worm studies. However, increasing active PLCβ1 increases activated CREB (p-CREB) levels. Both active and inactive PLCβ1 result in significantly higher levels of PSD95 higher nuclear, regardless of Gαq stimulation. Our results suggest that higher levels of PLCβ can suppress aversive associative memory by suppressing CREB levels.

The Gαq protein/phospholipase Cβ (PLCβ) signaling system is intimately involved in neuronal function. This pathway transduces signals from neurotransmitters such as acetylcholine and serotonin to increase intracellular calcium. Binding of these agents to their specific receptors to activate Gαq/PLCβ initiates a series of reactions that increase intracellular calcium levels and control neuronal plasticity (1, 2, 3, 4, 5). PLCβ is specifically associated with learning, memory, responses to emotional stress and reward, reality perception, and memory consolidation. These mechanisms are thought to depend on the ability of PLCβ to initiate increases in intracellular calcium. However, our recent work has found that PLCβ can operate independent of Gαq to regulate translational processes through events carried out by an atypical cytosolic population (1). These studies lead to the suggestion that the impact of PLCβ on neuronal functions may involve calcium-independent as well as calcium-dependent processes.

PLCβ has been found to play a key role in neuronal cell differentiation in both cultured and primary neuronal cells. When cells are induced to differentiate, a rapid and pronounced increase in PLCβ levels occurs that precedes an increased expression of Gαq (2). This increase in PLCβ is absolutely necessary to transition the cell to the differentiated state and downregulating PLCβ but not Gαq ablates differentiation. In differentiated neuronal cell lines (PC12, SKNSH, NeuroT2 and astrocytes), PLCβ is required to maintain the differentiated state (3) and reducing PLCβ levels causes cells to return to the stem state. We have recently shown that overexpression of catalytically active or inactive PLCβ in undifferentiated PC12, NT2 and HEK293 cells induces cell differentiation through a mechanism independent of PLCβ′s catalytic activity (4). Our data suggests that this mechanism may involve the ability of the cytosolic population of PLCβ1 to modulate nuclear localization of the transcription factor EGR-1 (early growth response protein-1). Like PLCβ, EGR-1 is associated with neuronal activity (5), and when cells are induced to differentiate through the addition of agents such as nerve growth factor, retinoic acid or AraC, EGR-1 promotes the transcription of genes associated with the differentiated state, including its own gene (6). Additionally, the promoter region of the EGR-1 gene contains cyclic adenosine 3′, 5′-monophosphate (cAMP) response elements (CRE) which can be occupied by members of the cAMP-element response binding (CREB) protein family (7). CREB, which plays a key role in learning and memory formation, is activated by calcium (8). By regulating the transcriptional activity of EGR-1 through a calcium-independent mechanism, and by regulating the activity of CREB in a calcium-dependent manner, PLCβ has the potential to impact neuronal function in multiple ways. Here, we have begun to delineate the two roles of PLCβ in learning and memory using the nematode *Caenorhabditis elegans.*

*C. elegans* are an attractive model for studying the Gαq/PLCβ signaling pathway in learning because of the similarity of this pathway to that in mammalian cells (9, 10). *C. elegans* are a valuable model for neurodegeneration studies because of their organized neural system, short lifespan, genetic tractability, and optical transparency, allowing us to follow neural activation microscopically both at cellular and organismal levels (11). Previous work has found that activation of Gαq in *C. elegans* enhances memory and impedes age-related cognitive decline (12). Here, we have investigated the role of PLCβ in mediating learning and memory using *C. elegans*. We find that worms lacking PLCβ have enhanced responses to adverse conditions and that this effect may be mediated through extended CREB levels. In support of this idea, we show that in cultured PC12 cells, increased expression of either active or inactive PLCβ downregulates CREB and increases the expression of PSD95, whose levels are mediated by Egr-1. Taken together, our results correlate with the idea that PLCβ1 reduces the duration of adverse learning, in part, by controlling CREB levels through Egr-1.

## Results

### Loss of PLCβ1 impacts short-term negative associative memory formation

**Figure 1:**
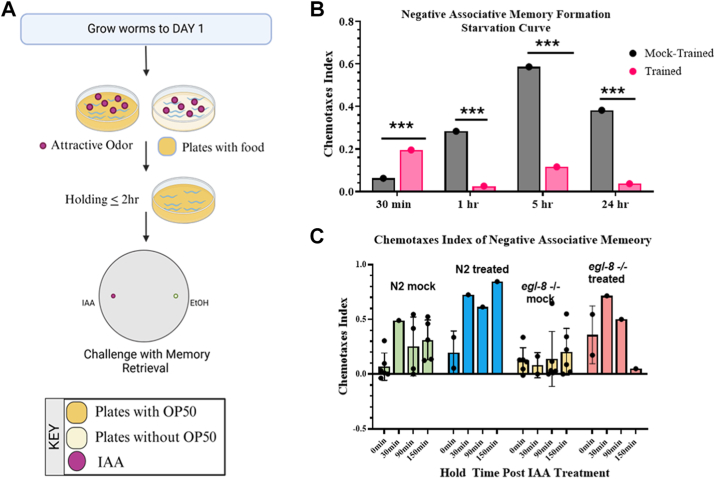
**Negative associative memory formation in wildtype and egl-8^−/−^mutant worms.***A*, diagram of the short-term negative associative memory paradigm used in these studies where worms were trained and starved for 30min-48 h by exposing worms to IAA (1:100) during the absence of OP50 *E. Coli* on an NGM plate. After training, worm’s chemotaxis index was analyzed after 1 h and counted manually. *B*, comparison of the chemotaxis index of Day 1 adult wild type N2 worms trained in the negative associative memory paradigm at different starvation times. For all conditions, n = 100 to 200 for one independent experiment, and ∗∗∗*p* < 0.001. *C*, differences in chemotaxis index in Day 1 adult wildtype and *egl-8*^*−/−*^mutant worms where after training in a negative associative memory paradigm, worms were placed on chemotaxes plates or moved to “holding” plates seeded with OP50 for 30 min, 90 min, or 150 min. Worms were analyzed after 1 h of chemotaxes and counted manually. Chemotaxis index values were visualized using GraphPad Prism and analyzed using an ordinary two-way Anova with Šídák's multiple comparisons test. For all conditions, n = 100 to 200 and 1 to 6 independent repeats were conducted (N = 1–6).

We determined whether PLCβ impacts memory formation in the model system *C. elegans*. Although the hermaphrodite *C. elegans* only has 302 neurons, whose connectivity has been mapped (10, 13), these small organisms can be trained in a memory paradigm (Fig. 1*A*) (9, 12, 14, 15). We assess training using a chemotaxis assay that determines movement of worms towards or away from a stimulant. Worms that cannot sense the stimulant exhibit random movement and are given a chemotaxis value of 0, whereas full movement towards or away from the stimulant are given values of +1 or −1.

For these studies, we synchronized wild-type (N2) worms at Day 1 adult, and trained them to associate an attractive odorant, isoamyl alcohol (IAA), with a negative experience (starvation). Control worms were placed on plates with either a bacterial food source (OP50) or plates without a bacterial lawn. Worms were then exposed to either EtOH treatment as a form of mock treatment or IAA by placing the odor on the lid. After training, we determined whether the worms avoided IAA using a chemotaxis plate spotted with the exposure odorant for 30 min, 1 h, 5 h, or 24 h post exposure (16). In Figure 1*B*, we show that mock-trained worms were attracted to IAA after 1 h, 5 h, and 24 h, whereas trained worms displayed strong repulsion values after 1 h up to 24 h starvation.

To determine the impact of PLCβ on associative memory formation, we compared adverse learning for wild-type (N2) worms and *egl-8*^*−/−*^(PLCβ^−/−^) mutant worms in which the mature enzyme is not produced (17). Again, Day 1 adult worms were collected and trained on plates with/without food and exposed to IAA or EtOH as a control. After 30-45 min of training, worms were assessed for their repulsion to IAA (0 min) or placed on plates seeded with nutrients (OP50). After 30 min, 90 min, and 150 min of holding, worms were placed on chemotaxes plates and assessed for repulsion (Fig. 1*C*). For wild-type mock-trained worms, chemotaxes values remained elevated 30 min and 90 min after training and reached the highest attractive values at 150 min post-treatment, as expected. In comparison to wild-type trained worms, chemotaxes index values of *egl-8*^*−/−*^mutant worms were much lower and remained low at 150 min post-training. Mock-trained *egl-8*^*−/−*^mutant worms showed chemotaxis but at a slightly reduced level as wild type, indicating that PLCβ contributes to attractive behavior. Trained *egl-8*^*−/−*^mutant worms show very little chemotaxes even after 150 min. Taken together, these data suggest that PLCβ is required for forming and sustaining negative associative memory.

### Sensory neurons sustain integrity and morphology during negative associative memory formation

**Figure 2:**
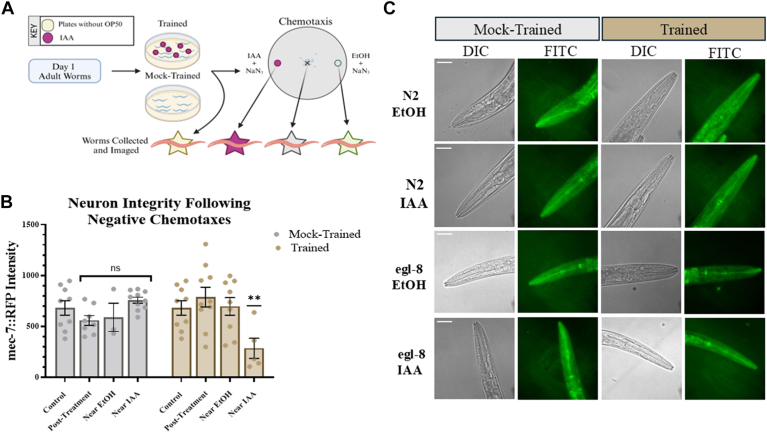
**Neuronal integrity and morphology during short-term negative memory formation.***A*, assay workflow for neuronal integrity and morphology analysis points during negative associative memory. Image created with BioRender. *B*, mechanosensory neuron integrity during short term negative chemotaxes. Adult Day 1 worms tagged with mec-7::RFP in mechanosensory heard neurons were collected and placed in a negative associative memory paradigm. Worms were trained and allowed to chemotax for 1 h, then collected and separated by location (*i.e.*, near origin, near IAA or near EtOH). Fluorescent intensity values were visualized using GraphPad Prism and analyzed using an ordinary two-way Anova with Tukeys multiple comparisons test where “ns” refers to non-significance, (∗∗) represents *p* < 0.01, and (∗∗∗) represents *p* < 0.001. For all conditions, n = 3 to 10 and two independent repeats were conducted (N = 2). *C*, representative confocal images of FITC-treated worms after negative associative memory paradigm. Adult Day 1 wildtype N2 and *egl-8*^*−/−*^mutant worms were treated with FITC by exposure to 0.1 mg/ml FITC on a seeded OP50 plate overnight to assess touch-neuron morphology. Post-treatment worms were placed in a negative associative memory paradigm and collected at different locations to assess neuron morphology. Images were taken at 25x where bright-field and 488 nm excitation images are shown. Scale bars are 20um.

Egl-8 is expressed in almost all *C. elegans* neurons (18) and thus its absence may promote dysfunction of neuron signaling. This possibility was tested by collecting Day 1 adult worms and measuring the short-term negative associative memory paradigm described in Figure 1*A*, at different times. Again, worms were trained on plates without OP50 to induce starvation, and after 1 h of chemotaxes, worms were collected either near the attractant, IAA or near the control, EtOH (Fig 2*A*). No paralytic was used during these assays to ensure that neuron integrity and morphology were not impacted.

*C. elegans* tagged with mec-7::RFP were imaged to assess neuron integrity. Mec-7 marks beta-tubulin structures in the mechanosensory neurons found in the head which can be visualized by the attached red fluorescent protein (RFP) (19, 20). Tubulin mec-7 is abundant in the touch receptor neurons that respond to external stimuli and internal forces generated during locomotion since these involve microtubule structures that depend on mec-7 for their formation and function (20). Thus, mec-7 expression serves as a direct readout to neuron integrity and normal neuronal function.

In Figure 2*B*, we show that mock-trained worms did not display significant changes in mec-7 fluorescence after deployment of the memory paradigm. For trained worms, we see relatively similar mec-7 fluorescence values for control, post-treatment, and those near EtOH. Interestingly, the worms found near IAA show a significant drop in mec-7 fluorescent signal, suggesting that the worms that did not form strong associative memory have lower integrity values.

We then collected wild-type N2 and *egl-8*^*−/−*^mutant worms near the attractant or control (Fig. 2*A*) and stained their touch neurons with FITC, which marks mechanosensory neurons in the head (Fig. 2*C*) (21, 22). Note that amphid and phasmid neurons in *C. elegans* have the capability to take up lipophilic dyes such as FITC and although the exact mechanism is not well understood, it is presumed that dye uptake is correlated with aspects of neuronal function. For both trained and mock-trained worms, there were no striking morphological differences observed for either worm strains. This result suggests that the observed lack of associative memory formation in egl-8−/−mutants is not due to loss of neuron integrity or large-scale abnormalities.

### Calcium remains elevated during negative associative memory paradigm

**Figure 3:**
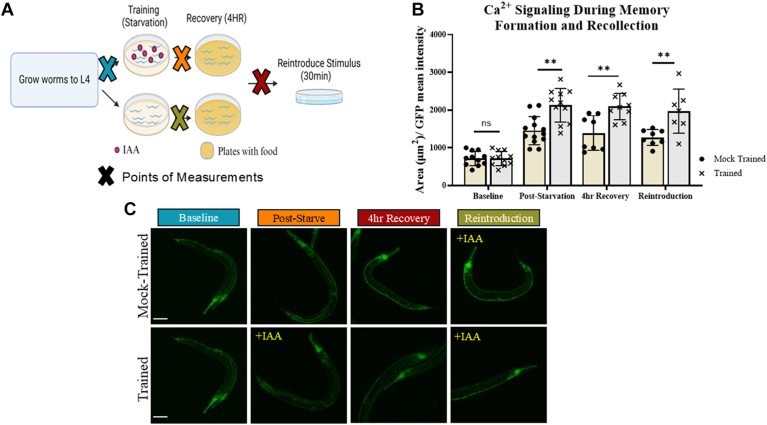
**Calcium signaling during negative associative memory paradigm.***A*, calcium analysis time points during the short-term negative associative memory paradigm where the colored “X”s indicate collection points. Image made with BioRender. *B*, fluorescent intensity values by area (Area of worm (um^2^)/mean fluorescence intensity) were visualized using GraphPad Prism and analyzed using an ordinary two-way Anova with Šídák's multiple comparisons test where “ns” correlates to non-significance, (∗∗) represents *p* < 0.01, and (∗∗∗) represents *p* < 0.001. For all conditions, n = 8 to 13 and two independent repeats were conducted (N = 2). *C*, representative confocal images of whole worms taken at 25x. Scale bars are 100 um.

Movement of *C. elegans* with IAA stimulation is mediated in part through calcium signals generated through the Gαq/PLCβ pathway (23). To better understand the data in Figure 1, we followed calcium signals that may be modulated by Gαq/PLCβ during associative memory formation and consolidation. Specifically, we measured calcium levels at different points of the learning paradigm to identify which steps are associated with strong calcium signals. In these studies, L4 worms tagged with a calcium reporter (GCamp), whose signal in neuronal cells predominate, were deployed on a negative paradigm (Fig. 3*A*) where GCamp in neurons predominate. Once training was complete, worms were allowed to recover for 4 h on plates seeded with OP50 and then exposed to IAA again for 30 min to view calcium signals associated with the reintroduction of the training stimulus. Intracellular calcium levels were measured before treatment (baseline), post-training, post-recovery, and upon reintroduction (Fig. 3*A*). We find that mock-trained and trained worms both show increased calcium levels (relative to the worm’s size) at every step of the deployed paradigm when compared to baseline (Fig. 3*B*). Additionally, trained worms show a significant increase in calcium signaling and usage as compared to mock-trained worms, indicative of greater PLCβ signaling output (Fig. 3, *B* and *C*). Higher GCamp signals in N2 worms reflect the contribution of egl-8 to basal calcium levels. We note that *Egl-8*^*−/−*^mutant worms did not survive the experiment which we attribute to the reduced resilience of these mutants to stress because of the ability of Egl-8 to mediate stress responses through mTORC-1 signaling (see (24)).

### Changes in CREB and EGR-1 levels with learning

**Figure 4:**
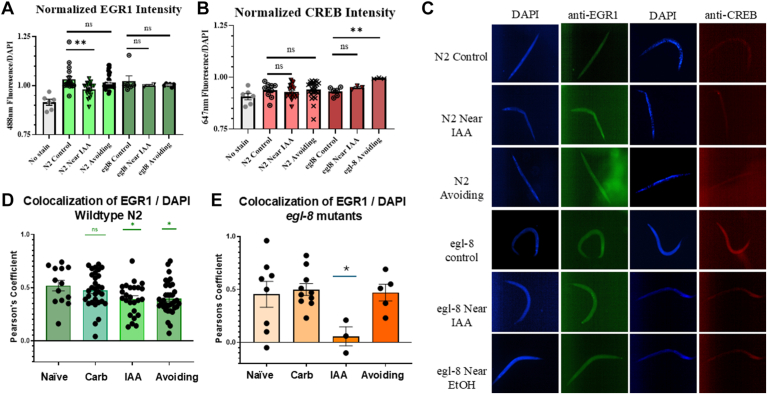
**Levels of EGR-1 and CREB after negative associative memory formation.** Wildtype N2 worms and *egl-8*^*−/−*^mutant worms were collected and placed in a negative associative memory paradigm. Worms were allowed to chemotax for 1 h and collected either near origin, near IAA or near EtOH, and then probed with (*A*) anti-EGR1 and (*B*) anti-CREB antibodies followed by fluorescently conjugated secondary antibodies (see Experimental procedure). Fluorescent values of CREB and EGR-1 were normalized to DAPI. Fluorescent intensity values were visualized using GraphPad Prism and analyzed using individual t-tests, where “ns” correlates to non-significance, (∗) represents *p* < 0.1, (∗∗) represents *p* < 0.01, (∗∗∗) represents *p* < 0.001, and (∗∗∗∗) represents *p* < 0.0001. For all conditions, n = 3 to 14 and four independent repeats were conducted (N = 4). *C*, Representative confocal images of head neurons taken at 10x. DAPI, anti-EGR-1 and anti-CREB are shown. Scale bars are 20um. *D* and *E*, Pearson’s coefficient showing colocalization between EGR-1 or CREB and DAPI in *w*ildtype N2 and *egl-8*^*−/−*^mutant worms under control (naïve) and Gαq stimulation (1 mM carbachol exposure for 30 min) Fluorescent intensity values were visualized using GraphPad Prism and analyzed using individual t-tests, where “ns” correlates to non-significance and (∗) represents *p* = 0.01. For all conditions, n = 11 to 15 and two independent repeats were conducted (N = 2).

*C. elegans* employ CREB activity and transcription machinery for long-term and short-term associative memory through a mechanism involving EGR-1 (15, 25, 26, 27). To understand our learning data, we examined levels of CREB and EGR-1 in worms after the formation of negative associative memory (Figure 4). Wild-type worms were collected on Day 1 and placed through the negative associative memory paradigm (Fig. 2*A*). Once complete, worms were collected based on their avoidance behavior where non-learning ones were collected near IAA and ones that learned were collected near EtOH. After collection, worms were stained with primary EGR-1 and CREB (see Experimental procedure) and then stained with respective secondary antibodies before confocal imaging (Fig. 4).

Changes in EGR-1 levels differed between wild type and *egl-8*^*−/−*^mutant worms. In wildtype worms, there is a significant drop in EGR-1 levels in worms found near IAA that have not formed negative memory (Fig. 4*A*). EGR-1 levels for control worms were similar to worms where memory formed. In contrast, *egl-8*^*−/−*^mutant worms showed no significant differences in EGR-1 levels in those found near IAA or EtOH. These observations are consistent with the idea that PLCβ1 plays a role in modulating the extent of negative associative memory (see Discussion). In contrast to EGR-1, we did not detect differences in CREB levels for wild-type worms found near IAA or near EtOH. Interestingly, *egl-8*^*−/−*^mutant worms suggest an increase in CREB levels for both worms strains near IAA and EtOH, and a reduction in EGR-1 nuclear localization in worms exposed to IAA. This reduced EGR-1 nuclear localization may indicate that the surviving worms did not learn, which is a behavior consistent with the cytosolic, non-transcriptional activity of EGR-1 (28).

### Gαq activation/PLC relocalization strengthens associative memory formation

PLCβ has a plasma membrane population that mediates calcium signals and also a cytosolic population that binds proteins involved in protein translation (29). In cultured cells, overexpression of PLCβ levels initially promotes EGR-1 nuclear localization initiating differentiation in the first 12 h, which are followed by a reduction at longer times (4). Because the cytosolic population can shift to the plasma membrane upon Gαq stimulation, we attempted to assess the contribution of the cytosolic population by activating Gαq with carbachol and probed for differences in memory formation compared to pre-treated worms.

In preliminary studies, we assessed the impact of Gαq stimulation on the attraction of naïve worm to IAA or another attractant, diacetyl (DA) to determine whether carbachol impacted memory formation or chemosensation. To do this, synchronized Day 1 wildtype and *egl-8*^*−/−*^mutant worms were collected, washed, and exposed to either diacetyl or IAA on a chemotaxis plate. This same assay was repeated, but with the addition of carbachol exposure before chemotaxes. We see no significant changes in IAA or diacetyl attraction behavior for wild-type or *egl-8*^*−/−*^mutant worms implying that chemosensation is not impacted by prior stimulation (Fig. S1*A*). We then activated Gαq to relocalize PLCβ prior to training using the same negative associative memory paradigm deployed in previous experiments (Fig. 1*A*). *Egl-8*^*−/−*^mutants showed a large loss in chemotaxis with Gαq stimulation before training since these mutants do not recover locomotion because they cannot terminate or rebalance cholinergic signaling once it has been activated due to defects in IP_3_/Ca^2+^ signaling, DAG/PKC signaling mediated by PLCβ (30).

Estimating changes in CREB and EGR-1 levels by their immunofluorescence intensity relative to DAPI in worms exposed to Gαq stimulation (*via* carbachol) (see Fig. S2 for representative images), we find similar levels of EGR-1 in both wild-type and *egl-8*^*−/−*^mutant worms. Thus, prior Gαq stimulation does not appear to impact EGR-1 or CREB levels.

### PLCβ1 differentially regulates CREB abundance and activation

**Figure 5:**
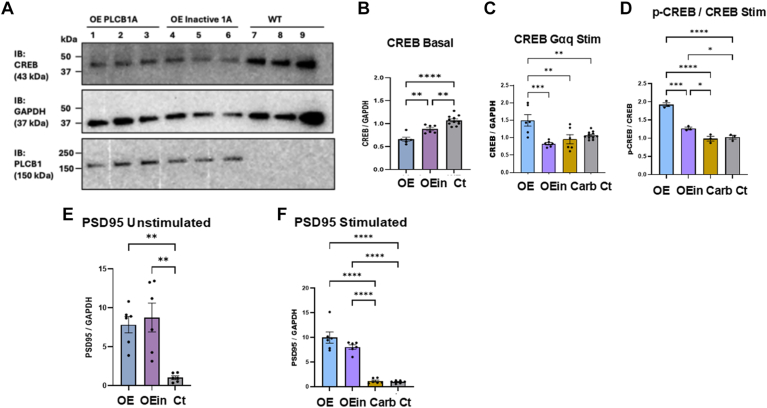
**PLCβ1 regulates CREB signaling and synaptic marker PSD95 expression in PC12 cells.***A* and *B*, immunoblots and quantification of CREB and PLCβ1 expression in PC12 cells overexpressing PLCβ1A (OE PLCβ1A), an inactive mutant (OE Inactive 1A), or wild-type (WT) control. CREB expression was significantly reduced in both OE PLCβ1A and OE Inactive 1A cells compared with WT cells. *C*, quantification of immunoblots (see Fig. S5 for full blots) of CREB and PLCβ1 expression following carbachol stimulation (+Carbachol). Carbachol treatment enhanced CREB expression in OE PLCβ1A cells compared with inactive mutant and WT controls. *D*, quantification of phosphorylated CREB (Ser133) relative to total CREB (n = 3). PLCβ1A overexpression significantly increased p-CREB levels upon carbachol stimulation compared with inactive mutant and WT cells. *E*, quantification of PSD95 expression (see Fig. S5 for full blots) with OE PLCβ1A and OE Inactive 1A cells compared with WT cells. *F*, immunoblots and quantification showing PSD95 and PLCβ1 expression after carbachol stimulation (+Carbachol) in each cell group. PSD95 expression remained unchanged after carbachol treatment. GAPDH served as a loading control. Data are presented as mean ± SEM (n = 6–12). Statistical analysis was performed using ordinary one-way ANOVA followed by Dunnett’s multiple comparisons test in GraphPad Prism. ∗∗*p* < 0.01; ∗∗∗*p* < 0.001; ∗∗∗∗*p* < 0.0001.

The results mentioned earlier suggest that PLCβ1 levels may impact the levels of EGR-1 and CREB. To test this idea, we switched our studies to cultured cells that enable easier assessment of protein levels. We first measured changes in CREB with over-expression of PLCβ1A, the most prevalent isozyme in neuronal tissue (31). Using western blotting, we find that under basal conditions, overexpression of wild-type PLCβ1A (designated OE in the figure) results in a significant reduction in CREB levels (Fig. 5, *A*–*D*). We then measured CREB levels after Gαq/PLCβ was stimulated with carbachol and compared these to samples where both active and inactive PLCβ1A (OEin) were overexpressed. This latter enzyme mutated two key catalytic residues (*i.e.* H331A and H378A) and was constructed based on previous studies demonstrating that these residues are essential for activity (32, 33) and Fig. S4). Notably, this mutant has been shown to regulate downstream signaling in fear memory formation in mice (34).

Before stimulation, we find that both CREB and activated CREB (*i.e.* p-CREB) are down-regulated regardless of whether PLCβ1 is active. After Gαq stimulation to increase intracellular calcium, cells transfected with active PLCβ showed a significant increase in CREB levels, but no changes were seen for the inactive mutant. Additionally, levels of activated CREB (p-CREB) were significantly higher when active PLCβ1 was overexpressed as compared to inactive consistent with calcium-induced CREB activation (Fig. 5*D*). Thus, PLCβ1 levels inhibit CREB production, but this inhibition can be reversed by increased calcium levels.

The data in Figure 5 are consistent with previous findings showing that increased cytosolic PLCβ levels promote Egr-1 nuclear localization (4) that may compete with CREB transcription ((4, 7) and see Discussion). To support this idea, we followed changes in the cellular level of PSD-95 since Egr-1 acts as a transcription suppressor of the PSD-95 gene. In the brain, it has been found that N-methyl-d-aspartate receptor activation induces Egr-1, which then binds to the PSD-95 promoter, reducing PSD-95 mRNA and protein (35). In Figure 5, *E* and *F*, we show that both active and inactive PLCβ1 enhance PSD-95 levels, and that carbachol stimulation reduces this increase (see Discussion). These results correlate well with the idea that PLCβ1 contributes to neural processes by both lipase-dependent and lipase-independent mechanisms.

**Figure 6:**
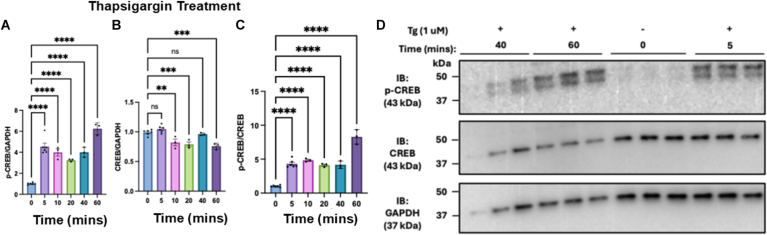
**Thapsigargin induces sustained CREB phosphorylation in PC12 cells.***A*, quantification of phosphorylated CREB (pCREB, Ser133) normalized to GAPDH following treatment with 1 μm thapsigargin (Tg) for the indicated times (0, 5, 10, 20, 40, and 60 min). *B*, quantification of total CREB levels normalized to GAPDH under the same treatment conditions. *C*, quantification of pCREB normalized to total CREB, representing CREB activation over time following thapsigargin treatment. *D*, representative immunoblots of pCREB (Ser133), total CREB, and GAPDH following thapsigargin treatment for the indicated time points. This assay measures CREB activation in response to intracellular Ca^2+^ elevation induced by ER Ca^2+^ store depletion. Data are presented as mean ± SEM (n = 3–6) from independent experiments. Statistical significance was determined using ordinary one-way ANOVA followed by Dunnett’s multiple comparisons test in GraphPad Prism. ∗∗*p* < 0.01; ∗∗∗*p* < 0.001; ∗∗∗∗*p* < 0.0001.

The data in Figure 5 show that overexpressing inactive or active PLCβ1 reduces CREB levels. The observation that active PLCβ1 has a more pronounced effect and that pCREB is greatly enhanced when cells overexpress active but not inactive PLCβ1 are stimulated by Gαq suggests the increased cellular calcium levels can also impact the level of CREB. To determine whether this is the case, we increased intracellular calcium levels using thapsigargin to empty calcium stores in the endoplasmic reticulum resulting in a large and sustained calcium increase (36). Thapsigargin treatment resulted in a 4- to 6-fold increase in pCREB (Fig. 6) which is far greater that that seen for calcium mobilization by Gαq stimulation of over-expressed PLCβ1. Additionally, this sustained increased calcium also resulted in a reduction in CREB at levels similar to that seen when inactive PLCb1 is overexpressed. These results suggest that PLCβ1 reduces CREB by a calcium-dependent mechanism and also by a lipase-independent mechanism.

**Figure 7:**
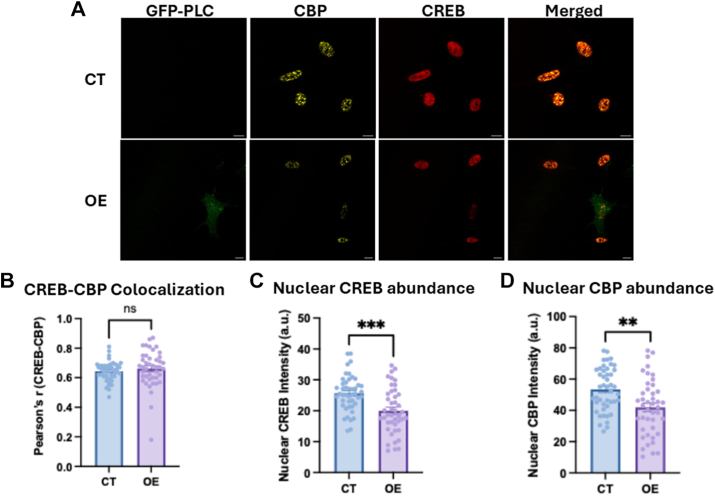
**Effect of PLCβ1 overexpression on CREB-CBP colocalization and nuclear CREB abundance.***A*, representative confocal images of undifferentiated (CT) and GFP-PLCβ1-overexpressing (OE) PC12 cells immunostained for CBP and CREB. GFP-PLCβ1 is shown in *green*, CBP in *yellow*, and CREB in *red*. Merged images are shown in the *right panel*. Representative images were acquired using identical imaging settings. Scale bar, 10 μm. *B*, quantification of CREB-CBP colocalization within nuclear regions of interest (ROIs) using Pearson’s correlation coefficient. Only GFP-positive cells were included in the OE group. Each symbol represents an individual nucleus (CT, n = 45; OE, n = 43). Data are presented as mean ± SEM and where ns is not significant. *C*, quantification of background-corrected nuclear CREB and CBP (*D*) fluorescence intensity. Fluorescence intensities were measured within manually defined nuclear ROIs, and local background fluorescence was subtracted from each measurement. Each symbol represents an individual nucleus (CT, n = 45; OE, n = 43). Data are presented as mean ± SEM. ∗∗∗, *p* < 0.001.

EGR-1 has been reported to oppose effects from CREB due to its ability to compete with the transcriptional activator CBP (CREB binding protein) (37). CBP is a transcriptional coactivator that binds only to the phosphorylated form of CREB (38) and is found in limiting amount in the cell. We followed changes in the level of CBP and its colocalization with p-CREB by immunofluorescence (Fig. 7). CBP has only been observed in the nucleus (39) and we only detect both CBP and CREB in the nucleus (see Fig. S7). Overexpressing active PLCβ results in a ∼25 to 50% reduction in the level of both proteins. However, their colocalization values remain unchanged. These data suggest that PLCβ levels do not greatly impact the activated state of CREB.

## Discussion

In this study, we investigated the role of Gαq/PLCβ signaling and its impact on memory consolidation and formation in *C. elegans*. *C. elegans* express homologs to Gαq (*egl-30*) and PLCβ (*egl-8*) proteins that have ∼40% identity primarily in their conserved structural domains and ∼70 to 80% overall homology to their mammalian counterparts (40). They also modulate intracellular calcium levels (41, 42, 43). *C. elegans* can be trained in memory paradigms involving key sensory neurons, odor responses and similar pathways to that of mammalian systems (14, 15, 44, 45). Here, we compared wild-type N2 worms with worms lacking PLCβ (*i.e. egl-8*^*−/−*^mutants). By associating a negative condition (starvation) with an attractant (IAA), we find that worms lacking PLCβ exhibit stronger repulsion for the stimulant as compared to wild-type worms, thus showing that the lack of PLCβ leads to increased negative associative memory formation. These results correlate well with previous studies showing that PLCβ1 knockdown in the medial prefrontal cortex of mice results in a heightened fear response and impaired memory extinction (46), as detailed below. We note that while PLCβ1−/− mice show behaviors similar to schizophrenia and post-traumatic stress disorder (47, 48, 49), their locomotion appears unchanged, which may indicate that PLCβ1 may have an additional impact on neuronal function besides calcium mobilization.

To understand the mechanism through which the absence of PLCβ increases the magnitude and duration of averse-associative learning, we focused on two transcription factors involved in learning and memory, EGR-1 and CREB. In mammalian systems, EGR-1 is essential for long term potential (5) and regulates the expression of PLCβ1, Gαq, PSD-95 and other genes involved in synaptic strength and plasticity (6) and may also impact transcription in a calcium-dependent manner (50). Blocking EGR-1 after retrieval disrupts the persistence of the memory, and like PLCβ1, EGR-1 plays a significant role in the development and persistence of PTSD (post-traumatic stress disorder) due to its involvement in fear-based memories (28). Additionally, CREB plays a key role in long-term memory formation in worms (15), where memory is mediated by the *crh-1* gene (*i.e.* the *C. elegans* homolog to CREB (51)). Interestingly, this same gene mediates massed learning, spatial learning, and short-term training models similar to the one we deployed. CREB has also been associated with long-term memory formation in other organisms such as *Aplysia* *and* *Drosophila* in addition to mammals (27, 52). Some of the first studies integrating a knockout CREB model reported deficits in spatial memory and long-term memory formation (53, 54). Collectively, *C. elegans* seem to share the negative implications of memory formation determined by diminished CREB, EGR-1 and PLCβ1 as in mouse models.

Our studies do not clearly distinguish between memory consolidation and extinction failure, or their underlying mechanism. Memory consolidation refers the formation of stable, long-lasting memory that is resistant to disruption whereas memory extinction occurs when repeated presentation of the stimulus leads to a decline in responding (see (55)). The curve in Figure 1*C* indicates early consolidation/maintenance of a short-term/early intermediate memory in both N2 and mutant worms. Consolidation is suggested because both worm types show normal acquisition at 0 min and at longer times, and maintenance is impaired, with *egl-8*^*−/−*^ returning to baseline while N2 remains aversive. At longer times (150 min), *egl-8*^*−/−*^ treated worms show chemotaxis close to its mock value whereas N2 treated is still negative. These results suggest that *egl-8*^*−/−*^ worms lose aversive memory faster than N2 who maintain a stable aversive memory over 150 min. Thus, egl*-8* activity is required for maintaining/stabilizing the negative associative memory.

To isolate the mechanism through which PLCβ is impacting memory, we first looked for alterations in neuronal morphology and calcium signaling. Changes in neuronal morphology and integrity were probed using established methods that involve a fluorescent-tagged tubulin protein (mec-7::RFP) and FITC-staining. We find that the only significant population to show a drop in mec-7 expression are worms found near the IAA stimulant, or worms that did not form a strong negative associative memory and that the integrity of touch neurons was similar in all groups. In another series of studies, we followed calcium signaling by confocal imaging at different points of the paradigm using worms tagged with the calcium reporter, GCamp::GFP (11). As expected, trained worms show significant increases at every point in the paradigm, showing the importance of calcium signaling in memory formation.

We tested whether PLCβ levels and activity influence CREB and EGR-1 levels in *C. elegans* using immunofluorescence. Although these measurements were experimentally difficult, we were able to conclude that the absence of PLCβ1 does not greatly impact the levels of EGR-1, while *egl-8*^*−/−*^mutant worms that show a strong memory formation have increased CREB levels. The idea that the impact of CREB becomes more pronounced without PLCβ is also consistent with the strong aversion of *egl-8*^*−/−*^mutant worms towards IAA. The few *egl-8*^*−/−*^mutant worms that did not form an adverse memory towards IAA appeared to have a much lower nuclear localization of EGR-1 that might not generate memory through EGR-1-mediated responses.

To better understand the impact of PLCβ levels on CREB/EGR-1, we switched to a model neuronal cell line PC12, which allows us to better quantify protein levels. Of course, we cannot directly compare these cultured cells with the organized neural network of *C. elegans*, but we hope that the basic cellular relationships between PLCβ1 and EGR1/CREB may help suggest potential mechanisms. In the nucleus, EGR-1 has been reported to oppose effects from CREB due to its ability to compete with the transcriptional activator CBP (CREB-binding protein) (37). While CREB activation is calcium-dependent and will depend on the plasma membrane function of PLCβ, how its levels and activation state depend on the cytosolic population of PLCβ, and on the extent of EGR-1’s nuclear localization is unknown.

In our worm studies, we compared wild-type worms to ones that do not express functional PLCβ and found little differences in EGR-1 levels. However, in cells, down-regulating PLCβ returns them to an undifferentiated state (3, 4). Thus, we compared undifferentiated PC12 cells, which express little PLCβ to cells where either active or inactive PLCβ is overexpressed. Previous studies in NT2 cells found that even though differentiation by AraC results in increased of ERG-1 levels (see Fig. S7 in (4)), differentiation by PLCβ results in a significant decrease in EGR-1. In PC12 cells, PLCβ overexpression results in a significant reduction of EGR-1 after differentiation. While it is possible that the decrease occurs at the transcriptional level, it is also likely that this occurs on the post-transcriptional level since PLCβ modulates the activity of RISC proteins (1, 56) and the formation of stress granules through a lipase-independent mechanism (57, 58). These studies indicate that the *C.elegans* neural network has the ability to maintain EGR-1 levels that are not present in isolated cultured cells.

We find that eliminating PLCβ in worms appears to increase CREB levels. This finding correlates with PC12 studies showing that overexpressing either active or inactive PLCβ1 significantly reduces CREB levels, with active PLCβ1 having a far greater impact. This latter effect is in-line with the decrease seen with thapsigargin treatment. However, when we stimulate the Gαq/PLCβ pathway and measure the ratio of active *versus* total CREB (*i.e.* p-CREB/CREB), we find that activated CREB levels are much higher, with active PLCβ correlating to the increased intracellular calcium. Comparison with thapsigargin-induced calcium elevation further indicates that calcium signals generated through distinct mechanisms differ in their ability to promote CREB activation. While receptor-mediated PLCβ signaling produces more localized and transient calcium increases, thapsigargin induces a more sustained and global elevation of intracellular calcium, resulting in a markedly stronger CREB phosphorylation response. Our data show that, while active PLCβ1 in turn activates CREB, the level of cellular PLCβ1 – either active or inactive - controls the level of cellular CREB, thus regulating the impact of learned behaviors. These cellular data are in accord with the observations that PLCβ1 regulates adverse associative learning seen here in worms and as previously reported in mice (34).

We qualitatively assessed changes in p-CREB using CBP. CBP binds only to the active form of CREB and this binding regulates the strength of CREB’s transcriptional activity (59). Even though PLCβ over-expression causes a similar reduction in both CREB and CBP, the proteins remain similarly colocalized suggesting that p-CREB and CBP remain complexed and presumably active with PLCβ over-expression and through PC12 cell differentiation. These data suggest that PLCβ expression does not greatly disrupt CREB transcriptional activity and this idea is currently being tested.

Because CREB and EGR-1 regulate transcriptional programs associated with neuronal differentiation and synaptic plasticity, we next examined whether PLCβ1 overexpression was accompanied by changes in a downstream synaptic protein. Specifically, we tested the ability of PLCβ1 to regulate PSD-95, whose expression inversely correlates with EGR-1 (60). Similar to PLCβ1 and EGR-1, PSD-95 production is associated with schizophrenia and autism (61). In response to specific stimuli, such as N-methyl-D-aspartate receptor (NMDAR) activation, EGR-1 acts a transcriptional repressor of PSD-95 (60) in a manner that is not directly associated with calcium levels. Interestingly, we find that increased PLCβ1 levels significantly increase PSD-95, and that this increase occurs regardless of PLCβ1 activity or Gαq activation. While these results suggest an alternate role of EGR-1 in regulating PSD-95 levels that is independent of NMDAR activation, salient for this work, it suggests that the cytosolic population of PLCβ plays a role in PSD-95 regulation and in turn synaptic maturation. Additionally, the large increase in PSD-95 seen with active and inactive PLCβ1over-expression is consistent with decreased EGR-1 and cytosolic PLCβ activity that may increase PSD-95 through both transcriptional and post-transcriptional mechanisms.

**Figure 8:**
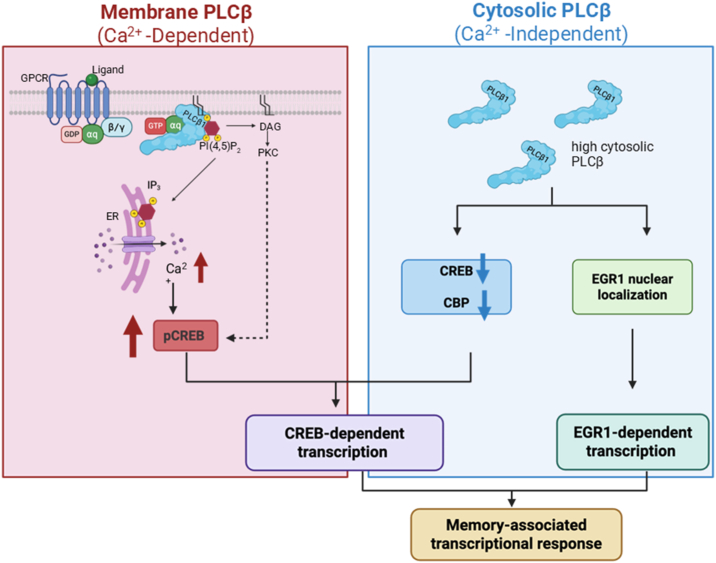
**Model of how PLCβ1 impacts learning.** Membrane-associated PLCβ1 promotes intracellular calcium signaling through the canonical Gαq/PLCβ pathway, resulting in CREB phosphorylation. In parallel, cytosolic PLCβ1 regulates CREB and CBP abundance and is associated with changes in EGR-1 nuclear localization. Together, these pathways may contribute to CREB- and EGR-1-associated transcriptional responses linked to memory-related gene regulation.

Putting together these results with previous work, we suggest that under basal conditions where there is a higher amount of PLCβ1 in the cytosol, both EGR-1 and CREB levels are lower as compared to the stimulated state. Upon neurotransmitter activation of Gαq, the cytosolic population of PLCβ shifts to the plasma membrane to bind Gαq. Activated PLCβ1 then generates calcium that promotes the production of p-CREB (Fig. 8). The formation of stress granules and increased RICS activity due to reduced cytosolic PLCβ1 cause a net reduction of CREB and EGR-1, increasing the amount of CBP/p-CREB complexes and transcriptional events (see (62, 63)). Studies are being carried out to support this model.

## Experimental procedure

### Plasmid construction

The rat eGFP-PLCβ1a construct was cloned into the pCDNA2.0 vector (a gift from Dr Catherine Berlot). Site-directed mutagenesis was performed to substitute histidines 331 and 378 with alanine using Q5 Site-Directed Mutagenesis Kit (E0554S, NEB) and Q5 High-Fidelity DNA Polymerase (M0491S, NEB).

### Cell culture

Rat pheochromocytoma cells (PC12; ATCC, CRL-1721.1) were cultured in Dulbecco’s Modified Eagle Medium (DMEM) supplemented with 10% heat-inactivated horse serum, 5% fetal bovine serum (FBS), and 1% penicillin–streptomycin (Gibco, Grand Island, NY, USA). Cells were maintained at 37 °C in a humidified incubator with 5% CO_2_.

### Transfection and carbachol stimulation

Cells were seeded into 6-well plates and transfected with either wild-type or H331A/H378A mutant eGFP-PLCβ1a plasmids using Lipofectamine 3000 (Invitrogen). At 72 h post-transfection, cells were stimulated with 5 μm carbachol, rested for 30 min or one hour, re-stimulated, rested for another 30 min, and collected for Western blot analysis (64).

### Thapsigargin (Tg) treatment

For calcium store depletion experiments, cells were treated with 1 μM thapsigargin (Tg) (CST, 12,758) for the indicated time points (0, 5, 10, 20, 40, and 60 min). Thapsigargin was prepared as a stock solution in DMSO and diluted in culture medium immediately before use. Control cells received an equivalent volume of vehicle (DMSO).

### Western blotting

Cells were lysed in ice-cold Triton X-100 lysis buffer (with protease inhibitors) for 30 min at 4 °C. Lysates were centrifuged at 15,000*g* for 5 min at 4 °C to remove debris. Protein samples were mixed with 4 × Laemmli buffer (Bio-Rad, 1,610,747), boiled at 70 °C for 10 min, and separated on 4 to 15% TGX Stain-Free gels (Bio-Rad, 4,568,086). Proteins were transferred onto nitrocellulose membranes (Thermo Scientific, 88,018) at 30 V, 4 °C, overnight. Membranes were blocked in 5% non-fat milk for 1 h at room temperature, followed by incubation with primary antibodies overnight at 4 °C. Primary antibodies: anti-CREB (1:1000, CST, 9197), anti-pCREB ser133 (1:1000, CST, 9198), anti-PSD95 (1:1000, CST, 3450S), anti-PLCβ1 (1:1000, Santa Cruz, sc-5291), and anti-GAPDH (1:10,000, Abcam, ab181602). Membranes were washed with TBS-T, incubated with HRP-conjugated secondary antibodies in blocking buffer, and rinsed again. The following secondary antibodies were used: m-IgGκ BP-HRP (1:2000, Santa Cruz, sc-516102) and anti-rabbit IgG, HRP-linked (1:10,000, Thermo Fisher, 31,460). Membranes were stripped using Restore PLUS stripping buffer (Thermo Fisher, 46,430) for 10 min at 37 °C. Signal quantification and image analysis were performed using ImageJ and GraphPad Prism 9.

### Calcium Measurements

Cells were seeded onto poly-D-lysine–coated glass-bottom dishes (MatTek Corporation). For single-cell calcium measurements, cells were incubated with 5 μm Calcium Green (Thermo Fisher Scientific) in Hank’s Balanced Salt Solution (HBSS) for 45 min at 37 °C, followed by two washes with HBSS to remove excess dye. Live-cell imaging was performed using a Zeiss LSM 510 Meta confocal microscope with time-series acquisition. Cells were stimulated with 5 μm carbachol, and images were collected for up to 5 min post-stimulation. Fluorescence intensity changes were quantified using ImageJ software.

### *C. elegans* strains and maintenance

All worm strains were obtained from *Caenorhabditis* Genetics Center (cgc.umn.edu). Strains, NM4397 (jsls973[mec-7p::mRFP + unc-119(+)]); MT1083 (n488(egl-8)); and QW1166 (zfIs42 [rig-3p::GCaMP3::SL2::mCherry + lin-15(+)]). Standard culture methods were used to maintain *C. elegans* (65). All strains were grown on nematode growth media (NGM) agar plates seeded with OP50 *Escherichia coli* (*E. coli*). The strains were maintained at 20 °C.

### Synchronization assay

Worms were synchronized to observe age-related functions using a typical bleaching protocol (66). Gravid worms were collected off NGM OP50 plates, washed with M9 Buffer (3*g* KH_2_PO_4_, 6*g* Na_2_HPO_4_, 5*g* NaCl, 1 ml 1M MgSO_4_, 1 ml 1M CaCl_2_, 1 ml of 5 mg/ml cholesterol in ethanol, to 1L H_2_0, sterilized by autoclave), and bleached to release eggs. Bleaching solution was made fresh (diH_2_0, 5% NaOCl (35% final volume), and 5N NaOH (20% final volume). Once released, eggs were spun down and washed with M9 buffer. Eggs were plated on fresh NGM OP50 plates and allowed to progress to L4 larvae. Once at an appropriate age, synchronized worms were moved to fresh OP50 plates and used for various assays.

### Carbachol stimulation

Carbachol was used to stimulate the Gαq subunit to exhibit stress (57, 67). Carbachol in powdered form was obtained from Sigma-Aldrich (carbamoylcholine chloride, Catalog #C4382) and dissolved in water to a final concentration of l mM at a volume of 25 ml. 1 ml aliquots were stored at −20 °C for long-term storage. Worms were washed with M9 buffer placed into a 15 ml centrifuge tube, allowed to sink to the bottom, then excess M9 buffer was removed. 1 ml of 1 mM carbachol was added to the centrifuge tube for 30 min at room temperature. Once stress was complete, carbachol was removed and worms were washed in M9 buffer and moved to a fresh OP50 plate for recovery periods at room temperature.

### Preparation of worms for imaging

For confocal microscopy worms were placed onto an agar pad using 2.5% noble agar on a cover slip with a paralytic agent for ease of viewing. To paralyze, worms were placed in 1uL of 100uM tetramisole hydrochloride (Sigma-Aldrich, Catalog #L9756) prepared in diH_2_0 or 1uL of 1M sodium azide prepared in diH_2_0. For each 1uL drop, 2 to 8 worms were placed before the drop was dry. Once worms were placed on the agar pad, another cover slip was placed on top to ensure worms stayed hydrated during imaging. For immunohistochemistry studies, worms were taken from the final wash (see Immunohistochemistry) and placed into a 35 mm petri dishes affixed with a poly-D-lysine treated glass-bottom (Mat-Tek cat#P35GC-1.5-14-C). Excess M9 buffer was removed and 100 μl of cooled 2.5% agarose was placed on top of worms. Once agarose was set, dishes were covered with 1 ml of M9 buffer to remain hydrated while imaging.

### Immunohistochemistry using *C. elegans*

For immunohistochemistry, a procedure was adopted from a previously established method (68). *C. elegans* were first synchronized using the synchronization protocol (see above) and collected at Day 1 with 1 ml of M9 buffer, spun down, and M9 was removed. Worm pellets were washed 3 times to remove any bacteria and then placed on ice for 3 min in 500ul of diH20. Fixative solution was prepared and 500uL was added to each pellet. Pellets were then placed in a liquid nitrogen bath for 1 min and then thawed using a water bath. This was repeated 2 times for a total of three freeze-thaw cycles. Worms then shook at low speeds for 1 h at room temperature.

Post fixation, worms were spun at 10,000 rpm for 1 min and fixative solution was removed. Worms were washed with Tris-Triton solution and Tris-Trion B-mercaptoethanol solution with 1 min 10,000 rpm spins in between washed. Worms then shook at low speeds in Tris-Trion B-mercaptoethanol solution for 1 h at room temperature. After shaking, worms were spun down gently (3000 rpm) for two minutes and 1 ml of borate buffer solution was added. Worms were then washed for 15 min each with borate buffer + DTT followed by borate buffer wash, borate buffer + peroxide followed by a borate buffer wash and then borate buffer and antibody buffer. Following last wash, borate-buffer and antibody buffer were removed and moved to new 1.5 ml tube with 1 ml of antibody buffer.

Worms were blocked with a 50:50 mixture of antibody buffer (68) and fetal bovine serum (FBS). Worms were shook for 1 h at room temperature. Blocking solution was then removed and replaced with primary antibody. Worms were probed with primary CREB (Santa Cruz, cat#sc-377154), or EGR1 (ThermoFisher Scientific, cat#PA5-80574) prepared in antibody buffer at a 1:250 dilution overnight at 4 °C on 2D shaker. Worms were washed x3 with antibody buffer and spun down between washes. Worms were then probed with Alexa Fluor 488 nm chicken anti-rabbit (ThermoFisher Scientific cat#A21441) or with Alexa Fluor 647 nm goat anti-mouse (ThermoFisher Scientific, cat# A-20990) prepared in antibody buffer at a 1:1000 dilution overnight at 4 °C on 2D shaker at low speed and protected from light. Worms were washed x3 with antibody buffer and spun down between washes. Once washed, worms were moved to fluorescent microscopy preparation (see above) or DAPI staining (see below).

### FITC-treatment

To stain amphid/phasmid neurons, lipophilic dyes can be used, the most common being Fluorescein isothiocyanate (FITC) (see (69)). First, a stock solution of FITC (TCI Chemicals, cat#3326-32-7) was prepared at 20 mg/ml in DMSO and aliquoted to 20 μL (stored at −20C). 20 μl of concentrated FITC (20 mg/ml) was added to 200 μl of M9 buffer in a 1.5 ml centrifuge tube. The diluted solution was added to a 60 mm NGM plate seeded with OP50 and allowed to sit for 2 h before beginning the assay (final FITC conc. = 0.1 mg/ml). Synchronized worms were collected, washed x3 with M9 to remove bacteria, and placed on FITC-treated plates for 24 h at 20C.

### DAPI treatment

Worms are fixed (see above) using hard fixation methods and then washed three times to remove residual fixative solution. Washed worms were then exposed to 100 ng/ml 4′,6′-diamidino-2-phenylindole hydrochloride (DAPI) in DPBS for 30 min under constant gentle shaking. Samples were washed three times using DPBS and moved to confocal imaging preparation.

### Confocal imaging

For immunofluorescent studies, confocal images of worms were obtained using a Nikon inverted confocal microscope in an ISS Alba System and NIS Elements software. Using a 10x objective, whole worms were imaged with 1s exposure time in the DIC, FITC, TRITC, and UV filter manually. For calcium studies, confocal images of worms were obtained using a Zeiss LSM510 Meta inverted confocal microscope. Worms head regions were captured using a x40 and x25 (water objectives) and acquired using singular and z-stacks. Single-frame images were acquired using a 1024 × 1024 pixel depth and 5s scan speed. Images were analyzed using ImageJ software and calculating the average fluorescence intensity based off region on interest (ROI) regions determined by bright-field images. Z-stack images collected 1 μm thick slices to capture the whole head region using a 560 × 560 pixel depth and 5s scan speed. Images were analyzed using ImageJ software by combining all slices and calculating the average fluorescence intensity based off region on interest (ROI) regions determined by bright-field images.

### Preparation of chemotaxis plates

Chemotaxes plates were prepared using NGM recipe (See cooled, solution was poured into 100 mm petri dishes and allowed to cool for 1 h at room temperature. Plates were stored at 4 °C for long-term storage. For chemotaxes the bottom of the plate was marked with two dots 7.0 cm apart and 0.75 cm from the edge of the plate. In the middle of the plate, 4.25 cm from the edge, an “X” was marked to represent the origin. Once labeled, 100 mm plate was flipped over and treated. On both spots, 1uL of 1M sodium azide was added. On the odorant spot, 1uL of 1:1000 DA or 1:100 was added on top of the sodium azide drop. On the other side, 1uL of 100% EtOH was added on top of the sodium azide drop. Plates were prepped 15 min before the chemotaxes occurred.

### Chemotaxis training

For chemotaxes assays, a procedure was adopted from previously established method (15, 44). *C. elegans* were first synchronized using the synchronization protocol (see above) and collected at Day 1 with 1 ml of M9 buffer, spun down, and washed twice with M9 buffer and once with diH_2_O. Once washed, worms were placed onto 60 mm OP50-seeded NGM plates or 60 mm unseeded NGM plates with a cut 200ul pipette tip to ensure worms integrity. Once dry, plates were treated with either 6 μl of odorant (1:1000 DA or 1:100 IAA) in a swirl pattern on the lid, then immediately placed upside down for 1 h at room temperature. Upon completion, worms were collected, washed twice with M9, and moved to Chemotaxes Assay (see below) or holding periods. For holding, worms were placed on OP50 seeded NGM plates for 30min-150 min and then collected and moved to Chemotaxes Assay (see below).

### Chemotaxis assay

Mock-trained or trained worms were placed onto prepared 100 mm chemotaxes plates (see above) with no more than 20 μl of M9 buffer with a cut 200uL pipette tip to ensure integrity of worms. Plates were left at room temperature to dry for 5-10 min and then flipped upside down and moved to a temperature (21C) and humidity-controlled (45%) room 1hr-2 h.

### Chemotaxis data collection and analysis

Upon the completion of the chemotaxes assay, worms near the odorant or EtOH control spot will appear straight-bodied and non-responsive due to the addition of 1M sodium azide paralytic. Plates were imaged using a Basler 2A4504-18ucPRO inverted camera. Once collected, PNG files were exported and manually counted using a tablet. Worms were considered near the odorant or the EtOH spot if they fell inside a circle around the spot with a diameter of 0.75 cm. Worms near the odorant, near the control spot, and all others were counted and used to calculate the chemotaxis index.

$$Chemotaxis\;Index=\frac{\left[\left(\#of\;worms\;near\;odorant\right)-\left(\#\;of\;worms\;near\;EtOH\right)\right]}{\left(Total\;number\;of\;worms\right)}$$

A chemotaxes value of 1.0 would indicate max attraction to odorant, where values of −1.0 would indicate max repulsion. All data were calculated using Microsoft Excel.

### Cellular immunofluorescence

PC12 cells were cultured on glass-bottom dishes and transfected with GFP-PLCβ1 where indicated. At 48 h after transfection, cells were fixed with 3.7% paraformaldehyde and permeabilized. Cells were then blocked with 5% normal goat serum for 30 min at room temperature and incubated overnight at 4 °C with primary antibodies against CREB and CBP. Primary antibodies included rabbit anti-CREB (1:100, CST, 9104) and mouse anti-CBP (1:100, CST, 7389). After washing, cells were incubated with Alexa Fluor 555 goat anti-rabbit IgG (1:500, Invitrogen, A-31572) and Alexa Fluor 647 goat anti-mouse IgG (1:500, Invitrogen, A-21236) for 1 h at room temperature. Nuclei were stained with DAPI (Roche Life Science). Fluorescence images were acquired using a Leica Stellaris eight point-scanning confocal microscope. Images used for quantitative comparisons between undifferentiated (CT) and GFP-PLCβ1-overexpressing (OE) cells were acquired using identical imaging settings, including laser power, detector gain, pinhole size, and scan acquisition parameters.

### Colocalization analysis

CREB-CBP colocalization was quantified using Fiji/ImageJ. Nuclear regions of interest (ROIs) were manually defined based on nuclear morphology and fluorescence signals. In GFP-PLCβ1-overexpressing samples, only GFP-positive cells were included in the analysis. Pearson’s correlation coefficient was calculated within nuclear ROIs using the Coloc2 plugin. Quantitative data were analyzed and plotted using GraphPad Prism 11. Each data point represents an individual nucleus (CT, n = 45 nuclei; OE, n = 43 nuclei).

### Quantification of nuclear CREB fluorescence intensity

Nuclear CREB fluorescence intensity was quantified using Fiji/ImageJ. Nuclear regions of interest (ROIs) were manually defined, and the mean CREB fluorescence intensity within each nucleus was measured. Background fluorescence was determined from a cell-free region within the same image and subtracted from the nuclear signal to obtain background-corrected nuclear CREB intensity. The same ROI selection criteria and background correction procedure were applied to all images. Quantitative data were analyzed and plotted using GraphPad Prism 11. Data are presented as background-corrected nuclear CREB intensity (arbitrary units, a.u.). Each data point represents an individual nucleus (CT, n = 45 nuclei; OE, n = 43 nuclei). The same nuclei were used for both colocalization and nuclear CREB intensity analyses.

### Statistical analysis

Data were analyzed with GraphPad Prism 10 statistical packages, which included the student’s *t* test and ordinary two-way analysis of variance (ANOVA).

## Data availability

All data are available upon request.

## Supporting information

This article contains supporting information.

## Conflict of interest

The authors declare that they have no conflicts of interest with the contents of this article.
