# Supplementary material for: Phospholipase Cβ regulates negative associative memory through calcium dependent and independent mechanisms
Source: J Biol Chem. 2026 Jun 29;302(8):113308. doi: 10.1016/j.jbc.2026.113308 (PMC13427652; doi:10.1016/j.jbc.2026.113308)
Supplement: Supplementary Material [file mmc1.pdf]

Supplemental

1 – Prior Gαq stimulation does not impact chemotaxis of N2 and egl-8 mutants to attractant

2 - Gαq stimulation does not impact chemotaxis or EGR-1 and CREB levels of Day 1 adult wildtype and *egl-8* mutant worms

3 – Representative images of worms immunostained with anti-EGR-1 and anti-CREB

4 – Calcium response of PC12 cells transfected with either active or inactive PLCβ1

5 – Full western blots of PLCβ1, EGR-1 and CREB from PC12 cells

6 - Full western blots of CREB and pCREB from PC12 cells after thapsigargin treatment

7 - Immunofluorescence images of CBP and CREB

Supplemental Figure 1

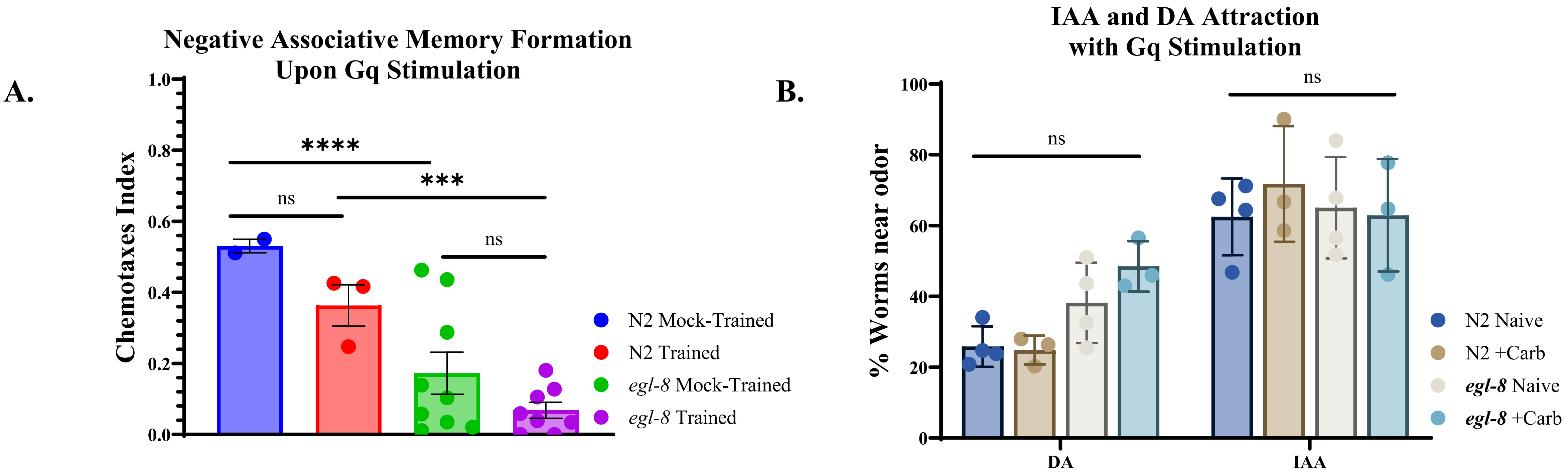

Prior Gαq stimulation does not impact chemotaxis to attractant. (**A**) Chemotaxis of Day 1 adult wildtype and *egl-8* mutant worms to attractant (IAA) after training in a negative associative memory paradigm were determined as described in the text. (**B**) Percent of worms that localize to either IAA (1:100) or DA (1:1000) and treated with 1M NaN3 to immobilize. The percent of worms near the attractive odor was analyzed after 1hr and counted manually. Data were visualized using GraphPad Prism. For all conditions, n= 100-200 and 1-6 independent repeats were conducted (N=3-4). Data are visualized using GraphPad Prism and analyzed using an ordinary two-way Anova with Šídák's multiple comparisons test.

Supplemental Figure 2

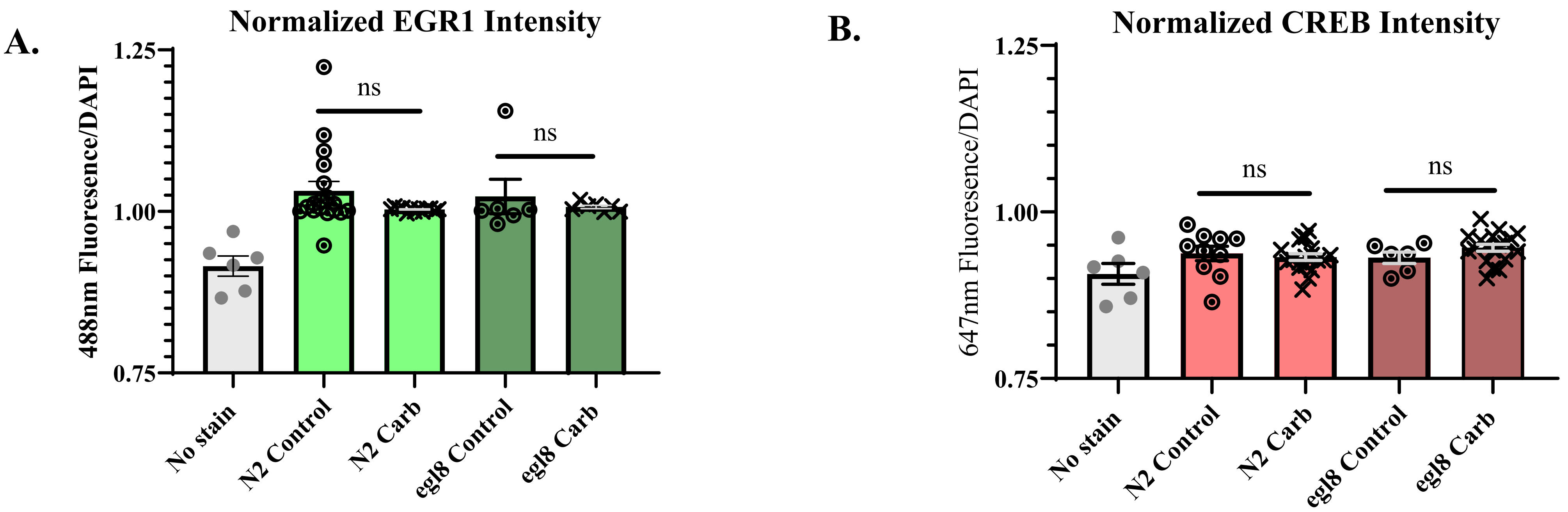

Gaq stimulation does not impact EGR-1 and CREB levels of Day 1 adult wildtype and egl-8 mutant worms. Worms were exposed to 1mM carbachol for 30 to stimulate Gaq before training, allowed to chemotax for and probed with **(A)** anti-EGR1 and **(B)** anti-CREB antibodies followed by fluorescently conjugated secondary antibodies (see Materials and Methods). Fluorescent values of CREB and EGR-1 are normalized to DAPI and visualized using GraphPad Prism and analyzed using individual t-tests, where “ns” correlates to non-significance. For all conditions, n= 3-14 and four independent repeats were conducted (N=4).

Supplemental Figure 3

Images of some of the samples in *Supplemental Figure 2* showing Day 1 N2 wild type and *elg-8* mutant worms under basal and Gαq stimulated conditions.

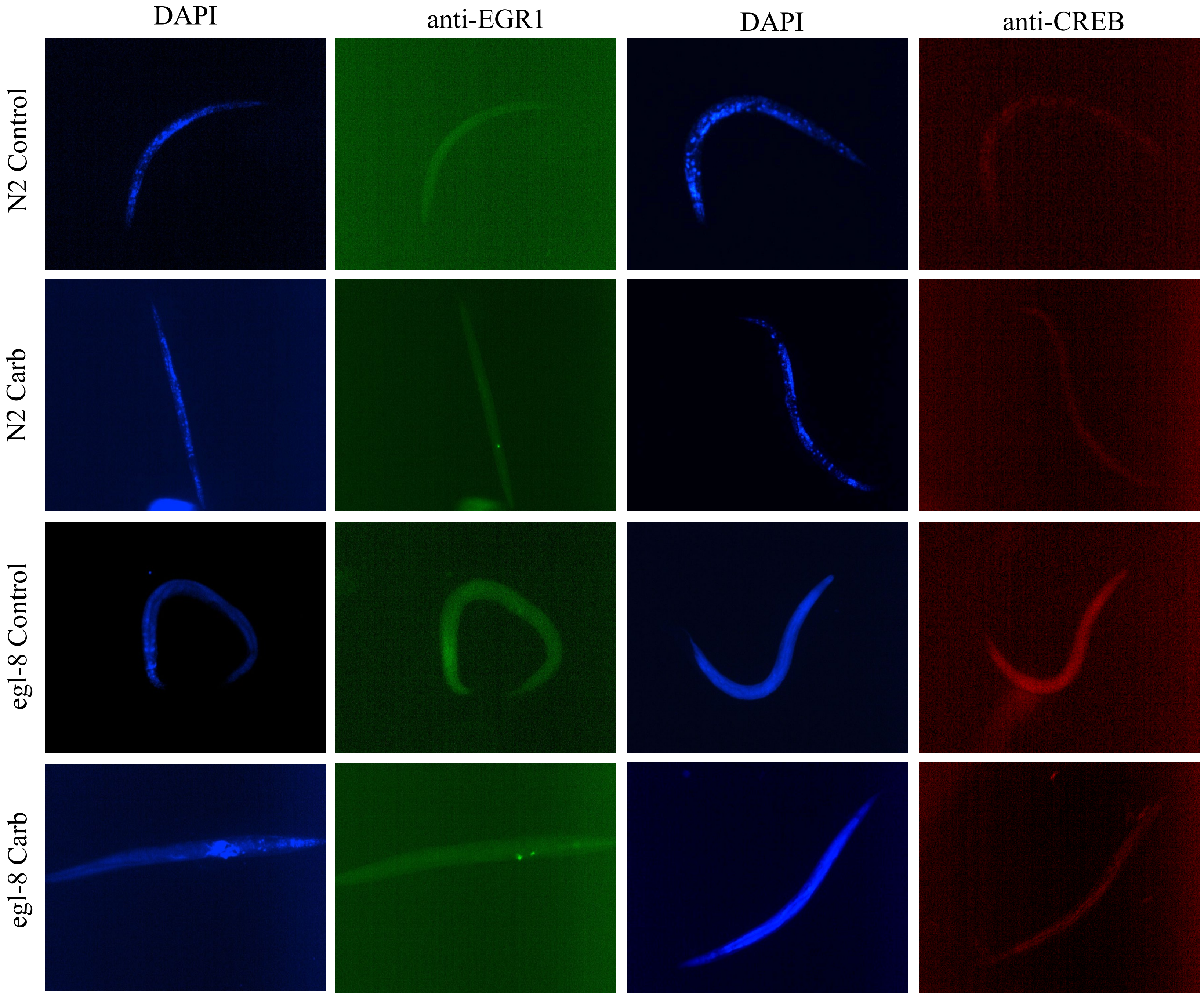

Supplemental Figure 4

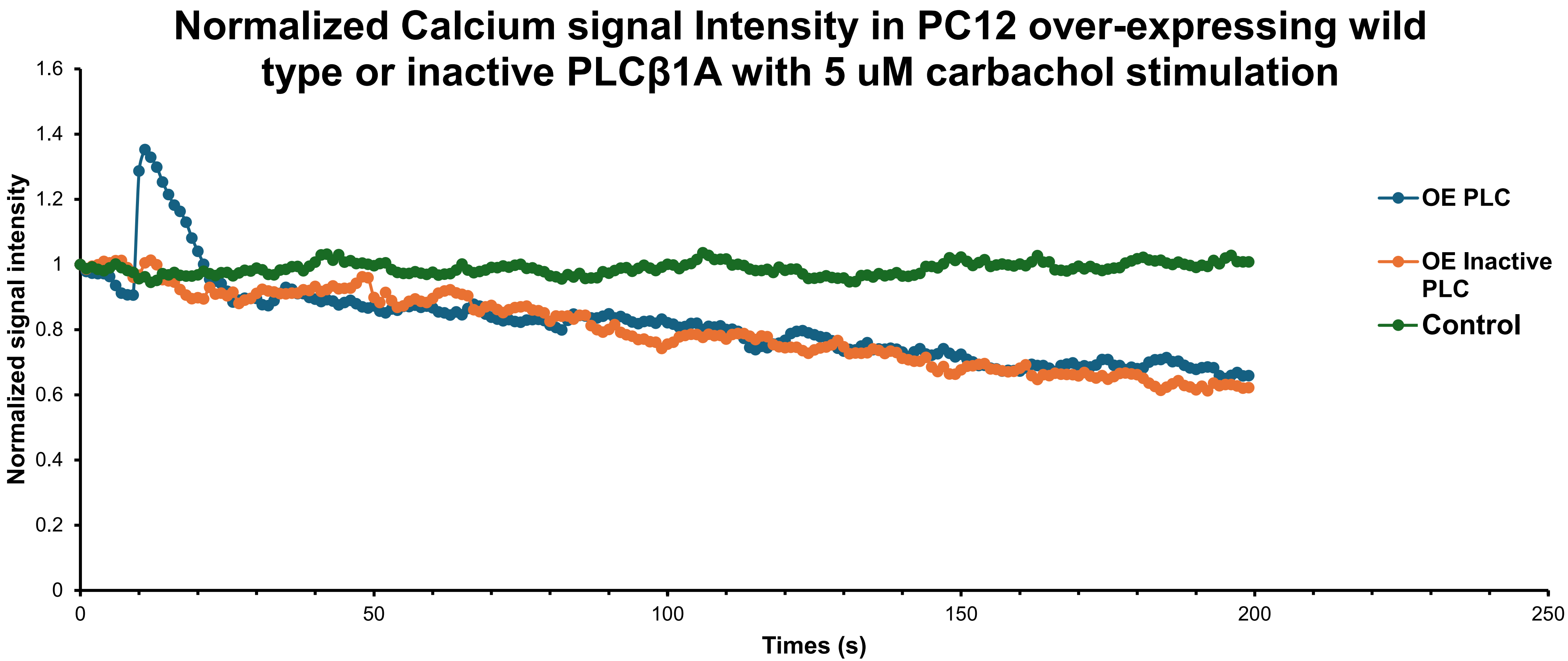

Calcium response of PC12 cells overexpressing wild type or inactive PLCβ1A. Normalized Ca^{2+} signal intensity was measured in PC12 cells overexpressing PLCβ1A an inactive mutant (Inact. + C), or untransfected control following 5 μM carbachol stimulation. A transient and robust increase in Ca^{2+} signal was observed in PLCβ1A-overexpressing cells, while inactive mutant and WT cells showed minimal response. Traces represent normalized mean signal intensity ± SEM from 4-6 individual cells analyzed as described under "Experimental Procedures."

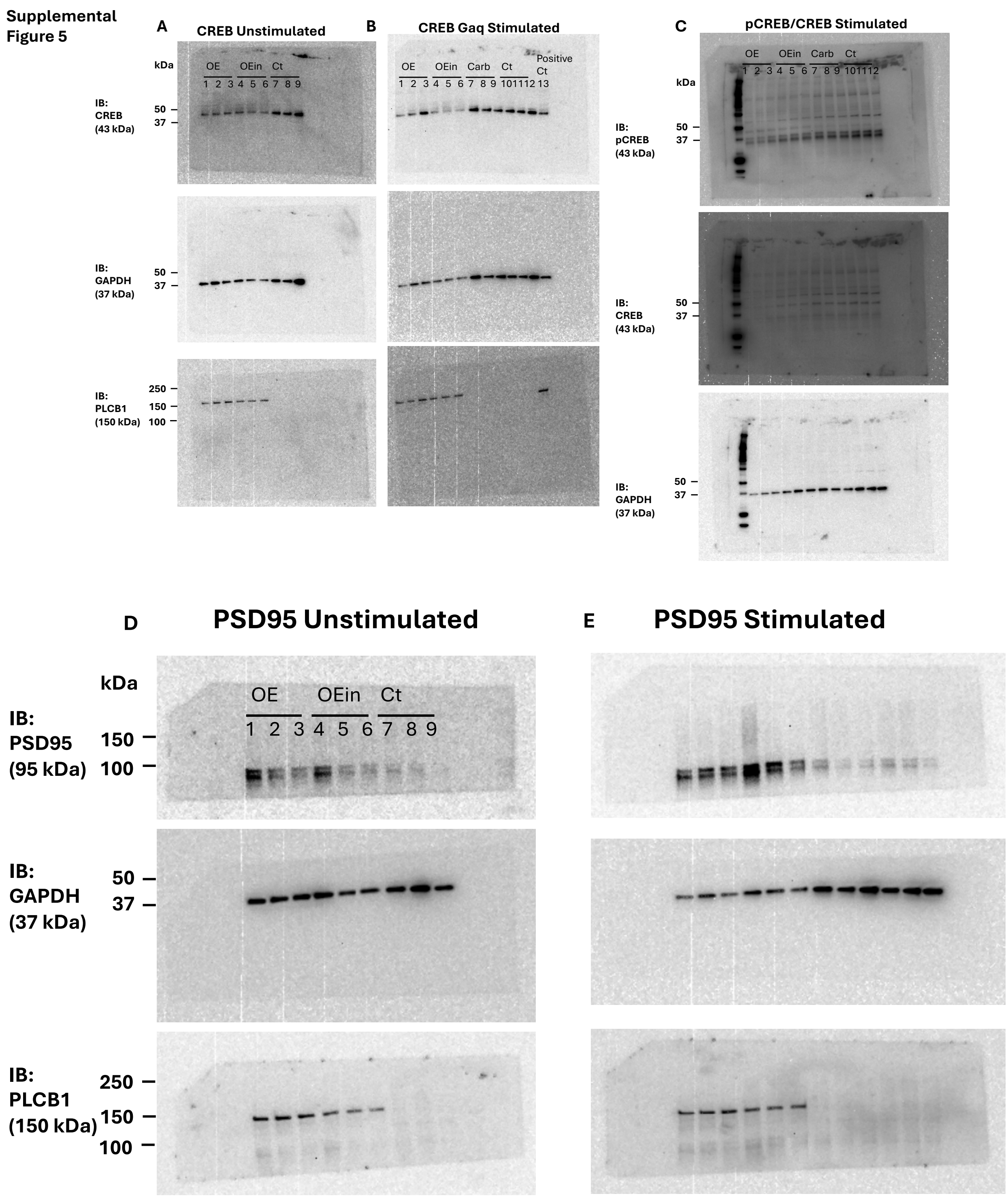

Supplemental Figure 5. PLCβ1 regulates CREB signaling and synaptic marker PSD95 expression in PC12 cells.

(A) Immunoblots of CREB and PLCβ1 expression in PC12 cells overexpressing PLCβ1A (OE), an inactive mutant (OEin), or wild-type (Ct) control.

(B) Immunoblots of CREB and PLCβ1 expression following carbachol stimulation (+Carbachol), a Gαq-coupled receptor agonist, in OE, OEin, and Ct groups.
(C) Immunoblots of phosphorylated CREB (pCREB) at Ser133 relative to total CREB after carbachol stimulation in OE, OEin, and Ct groups. The recovery time for this experiment after stimulation is one hour. This assay measures CREB activation
(D) Immunoblots of the synaptic marker PSD95 expression in unstimulated OE, OEin, Ct groups.
(E) Immunoblots showing PSD95 and PLCβ1 expression after carbachol stimulation (+Carbachol) in each cell group.
GAPDH served as a loading control.

Supplemental Figure 6

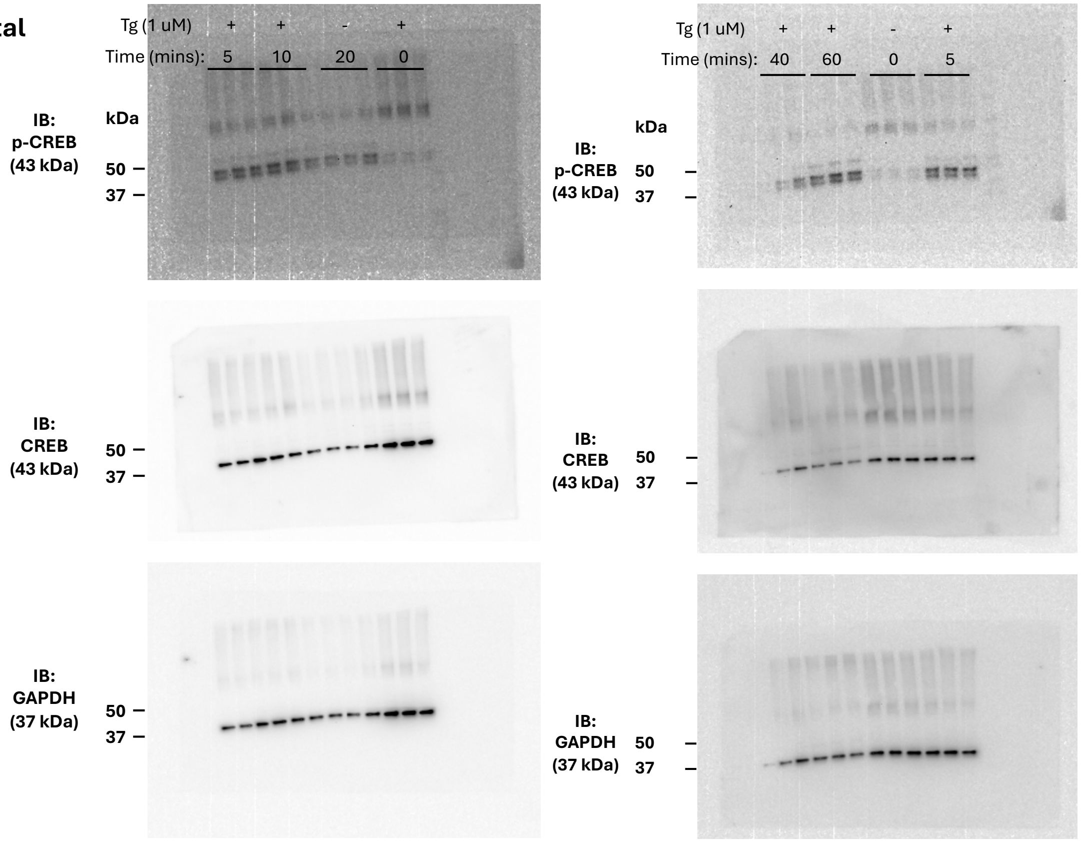

Supplemental Figure 6. Thapsigargin induces sustained CREB phosphorylation in PC12 cells. Left: Immunoblots of phosphorylated CREB (pCREB, Ser133) and total CREB following treatment with 1 µM thapsigargin (Tg) at early time points (5, 10, and 20 min) compared to the untreated control (0 min).

Right: Immunoblots of phosphorylated CREB (pCREB, Ser133) and total CREB following thapsigargin treatment (1 μM) at later time points (40 and 60 min), compared to the untreated control (0 min) and an early time point (5 min).
GAPDH served as a loading control.

Supplementary Figure 7

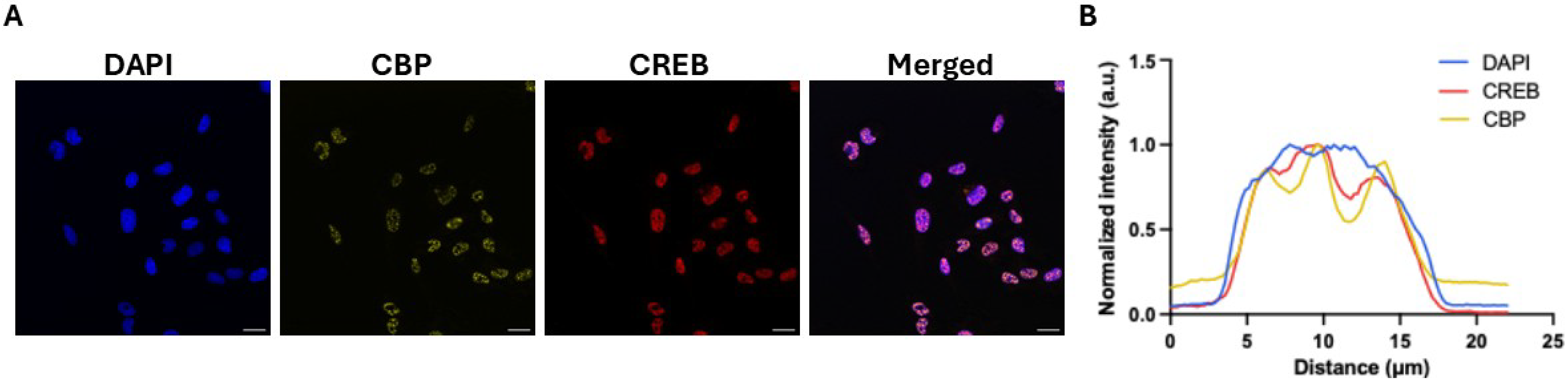

A - CREB and CBP are predominantly localized within the nucleus in PC12 cells. **(A)** Representative confocal images of undifferentiated PC12 cells stained with DAPI (blue), CBP (yellow), and CREB (red). Merged images demonstrate the localization of CREB and CBP relative to DAPI-positive nuclei. Scale bar, 10 µm. *See text for methods.*

(B) Plot profile analysis across a representative nucleus showing normalized fluorescence intensity profiles of DAPI, CBP, and CREB. Fluorescence intensities were normalized to the maximum value of each channel. The intensity peaks of CREB and CBP overlap with the DAPI-positive region, consistent with predominant nuclear localization of both proteins. Profile analysis was performed using Fiji/ImageJ. Representative images of undifferentiated PC12 cells stained with DAPI, CREB, and CBP were analyzed by drawing a rectangular region of interest (ROI) across representative nuclei. Fluorescence intensity profiles for DAPI, CREB, and CBP were extracted across the ROI using the Plot Profile function. For visualization, fluorescence intensities were normalized to the maximum value of each channel and plotted as a function of distance.
